# Exploring the relationship between personality traits, social appearance anxiety, and quality of life among nursing students

**DOI:** 10.1186/s12912-024-02665-7

**Published:** 2025-01-17

**Authors:** Rania Rabie El-Etreby, Eman Sameh AbdElhay, Nesma Ahmed Kamel, Samia Gamal Awad Hamed, Warda Elshahat Hamed

**Affiliations:** Psychiatric and Mental Health Nursing, Faculty of Nursing, Mansoura University, Mansoura, Egypt

**Keywords:** Nursing students, Personality traits, Social appearance anxiety, Quality of life

## Abstract

**Background:**

Nursing students represent a unique group that faces specific stressors. One of these stressors is social appearance anxiety, which can adversely affect the quality of life. Personality traits are strong predictors of coping with stress and anxiety. This study explored the relationship between personality traits, social appearance anxiety, and quality of life among nursing students.

**Methods:**

The study employed a correlational descriptive cross-sectional research design. One thousand twenty-one students from the bachelor program’s first, second, third, and fourth years participated in the study. The data was collected over three months from June to August 2024 by using four tools: the student's academic and socio-demographic profile, Novo Psych Five Factor Personality Scale (NFFPS-30), Social anxiety about appearance Scale (SAAS)., and The WHO Quality of Life Scale-Brief (WHOQOL-Brief).

**Results:**

The personality trait with the highest mean score is agreeableness (21.0 ±2.8), while neuroticism has the lowest mean score (18.4 ±4.3). (SAAS) results show that the majority of students (79.5%) experience low social anxiety levels. Additionally, (WHOQOL-Brief), general health, and social domains had the highest mean scores (58.2 and 53.2), compared to the physical domain, which scored the lowest at (40.6).

**Conclusion:**

The study concluded that all personality traits correlate significantly negatively with SAAS except neuroticism. QOL was positively correlated with all personality traits except neuroticism. SAAS and neuroticism traits of personality are strong negative predictors of QOL. Further research and interventional programs are necessary to detect students’ level of anxiety and their personality traits and improve their social appearance anxiety.

## Introduction

Personality is a combination of characteristics that affect how you think, feel, and behave. It includes dispositions, attitudes, and behaviors that manifest in interactions, combining inherent and acquired characteristics that distinguish individuals in their social environments [1, 2]. One of the most well-known theories of personality is the Five-Factor Model, also known as the Big Five personality traits. Openness, conscientiousness, extraversion, agreeableness, and neuroticism are the five key dimensions of this model, which Costa and McCrae refined in the late 1980s and early 1990s [2, 3]. Neuroticism reflects a tendency toward negative emotional states such as anxiety, worry, and sadness. Extraversion indicates how outgoing and social a person is. Openness involves a willingness to embrace new experiences and ideas. Agreeableness pertains to kindness, cooperation, and prosocial behaviors. Conscientiousness denotes a preference for organization, dependability, and goal-oriented behavior [4].

Research has shown that personality traits are influential factors in various types of anxiety [5]. Anxiety can severely disrupt daily life, leading individuals to avoid social interactions and isolate themselves. This is particularly pertinent in the context of social anxiety, which is the fear of receiving unfavorable judgment from others. Cultural studies indicate that social anxiety is a universal experience affecting individuals across cultures [6]. Furthermore, looking at these connections through the biopsychosocial model helps show how personality traits, biology, psychology, and social factors impact mental well-being.

Social appearance anxiety is when you worry about how others judge your physical looks [7]. Social expectations of beauty exacerbate these anxieties by making people self-conscious about their bodies and reluctant to interact with others [8]. For instance, studies have shown that social appearance anxiety affects approximately 30–40% of young adults in higher education, highlighting its prevalence and impact [8]. This type of anxiety is particularly pronounced during adolescence and early adulthood, a critical developmental stage for self-identity and social interactions [9, 10]. According to the World Health Organization (2020), quality of life is how a person sees their life, including their culture, values, and goals, as well as their health, education, and sense of belonging [11].

Nursing students have distinct challenges like academic demands, clinical training, and professional expectations. These factors can increase social appearance anxiety, particularly given the importance of maintaining a professional appearance in healthcare settings [12]. Previous studies have shown that feeling unhappy with your body and worrying about how you look can harm mental well-being, leading to more stress and poorer mental health [13]. Furthermore, understanding the dynamics of social appearance anxiety is crucial in nursing education, as it directly impacts students' ability to engage confidently in clinical settings, which is essential for effective patient care and professional development. However, there is a need for more studies investigating how personality traits impact these aspects for nursing students [14, 15].

Despite the recognized impact of personality traits on anxiety and quality of life, existing studies have primarily focused on general populations and have not adequately considered the unique experiences of nursing students [12]. Additionally, there is limited understanding of the specific personality traits that may worsen or alleviate social appearance anxiety and how these factors collectively impact nursing students' well-being and academic performance [16]. Recognizing these gaps is crucial for developing specific strategies to help improve nursing students' mental health and quality of life. Therefore, this study aimed to examine the relationships between personality traits, social appearance anxiety, and quality of life among nursing students. The main questions of this study were: What is the relationship between personality traits and social appearance anxiety among nursing students? How do different personality traits impact the quality of life of nursing students? Is there a significant relationship between social appearance anxiety and the quality of life among nursing students?

## Methods

### Study design and setting

This study employed a cross-sectional descriptive correlational research design to investigate relationships between personality traits, social appearance anxiety, and quality of life among nursing students. The research was conducted at the Faculty of Nursing, Mansoura University, under the direction of Egypt's Ministry of Higher Education and by Egyptian nursing education standards.

### Sample and sampling procedure

A convenience sample of 1021 students was selected based on the following inclusion criteria: willingness to participate in the study, first, second, third, and fourth year of bachelor's degree, absence of cognitive, mental, or physical disabilities, and have the right to withdraw from the study at any time without any consequences.

The sample size was determined using data from the literature [17], considering a 5% level of significance and an 80% power of study. The sample size can be determined using the following formula: n = $\frac{(\text{Z}1 - {\upalpha }/2)^{\wedge}2.\text{SD}^{\wedge}2}{\text{d}^{\wedge}2}$. Where Z1-α/2 = is the standard normal variate, at 5% type 1 error, it is 1.96, SD = standard deviation of variable, and d = absolute error or precision. So, n = $\frac{(1.96)^{\wedge}2.(6.97)^{\wedge}2}{(0.471)^{\wedge}2}$ = 841.3.

According to the formula above, 842 is the total sample size needed for the investigation. Our sample is statistically significant because its size of 1,021 was greater than the minimum of 842. Therefore, we are confident that our findings will be reliable and robust.

### Data collection tools

Various self-report measures were used to provide a comprehensive framework to investigate the relationship between personality traits, social appearance anxiety, and quality of life.

#### Tool I: the student's academic and socio-demographic profile

The socio-demographic data, including age, gender, marital status, family income, number of family members, birth order, relationship with other people, mother and father educational level, BMI groups, and frequency of physical exercise, are included in the first part of this tool. In contrast, the second part contains academic information such as GPA, academic level, and academic achievement.

#### Tool II: the Novo PsychFive Factor Personality Scale (NFFPS-30)

The NFFPS-30 is a brief self-report personality test that assesses the well-known five-factor model of personality [18]. A Likert scale ranging from 1 (extremely incorrect) to 5 (highly accurate) is used to grade each item; higher scores correspond to more significant levels on each subscale. Adults and older teenagers (ages 16 and above) can use it to compare personality traits to age and gender norms on the five factors of Openness, Conscientiousness, Extraversion, Agreeableness, and Neuroticism: the internal reliability of the NFFPS-30 was 0.82, 0.91, 0.89, 0.86, and 0.90 for Openness, Conscientiousness, Extraversion, Agreeableness, and Neuroticism respectively [18].

#### Tool III: the Social Anxiety About Appearance Scale (SAAS)

The 16-item SAAS was created to measure the participant’s anxiety regarding situations where individual appearance can be assessed [19]. Each item has a response option ranging from 1 (not at all) to 5 (very). The first item is reverse-coded before adding up the scores from all 16 items. (Reverse coding the first item means inverting its scale values), This adjustment is made to align the scoring of the first item with the direction of the others (i.e., higher scores indicating more significant anxiety). After reverse coding, the values from all items are summed to produce a final score that ranges from 16 to 80. Low anxiety is defined as 16–37, moderate anxiety as 38–59 and high anxiety as 60–80. With an α of 0.96, the original English version of the SAAS has shown strong internal consistency and reliability [20].

#### Tool IV: the WHO Quality of Life Scale-Brief (WHOQOL-Brief)

WHO (1997) [21] translated the QOL into Arabic to evaluate people's views on their culture and values and their objectives, standards, and concerns. The tool consists of 26 items, with 24 addressing four domains and two additional items assessing general health and overall quality of life. Physical domain (7 Items; 3, 4, 10, 15, 16, 17, 18), Psychological domain (6 Items; 5, 6, 7, 11, 19, 26), Social domain (3 Items; 20, 21, 22) and Environmental domain (8 Items; 8, 9, 12, 13, 14, 23, 24, 25). Each item within these domains is rated on a 5-point Likert scale, ranging from 1 (very dissatisfied) to 5 (very satisfied). Domain scores are calculated by summing the raw scores for the items within each domain and transforming these into a standardized scale ranging from 0 (indicating the lowest quality of life) to 100 (indicating the highest quality of life). Additionally, two general items assess the overall quality of life and general health, which are analyzed separately to provide an overall subjective evaluation. Higher scores reflect better-perceived quality of life, offering insights into specific aspects of an individual's well-being.

### Validity and reliability

The Novo Psych Five Factor Personality Scale** (**NFFPS-30) and The Social Anxiety about Appearance Scale (SAAS) have been translated into several languages, showing satisfactory reliability and validity. For the present study, the researchers translated the study tools of NFFPS-30 and SAAS into Arabic after obtaining permission from the original authors. Its content validity was evaluated by five psychiatric and mental health nursing specialists from Mansoura University's nursing faculty. The reliability of tools was tested using Cronbach’s alpha test, NFFS-30 = 0.804, SAAS = 0.949, and WHOQOL-Brief = 0.922.

### Data collection procedure

Before starting the main study, a pilot study was carried out to evaluate the feasibility of the research tools and procedures. This preliminary phase served as a trial run for the fieldwork, enabling the identification of potential challenges and obstacles that could arise during data collection. It also helped determine how long it would take to finish the questionnaire. The pilot involved 110 nursing students, representing more than 10% of the study sample. The insights gained from this pilot phase provided valuable feedback on the research techniques' applicability and ease of use. Based on these findings, minor adjustments were made to the sampling strategy and data collection tools to enhance the overall study design (see Table 1).

**Table 1:** Pilot study results

Aspect Evaluated	Details
**Sample Size**	110 nursing students (more than 10% of the study sample)
**Average Time**	20 min (range: 15–25 min)
**Purpose**	To evaluate the feasibility of research tools and procedures
**Key Outcomes**	- Identified potential challenges and obstacles during data collection
	- Determined approximate time needed to complete the questionnaire
**Reliability of Tools**	
**NFFS-30**	Cronbach’s Alpha = 0.80 (Acceptable)
**SAA**	Cronbach’s Alpha = 0.94 (Excellent)
**- WHOQOL-Brief**	Cronbach’s Alpha = 0.92 (Excellent)

The data was collected over approximately three months, from June to August 2024. The study utilized an online platform (Microsoft Forms) that included a comprehensive study description, an informed consent form, and questionnaires. The survey link was distributed to students via email and WhatsApp, depending on their preferred method of communication. Students interested in participating could review the study description and provide informed consent before completing the questionnaire. The surveys typically took around 10 to 15 min to complete.

### Statistical analysis

IBM SPSS software package version 23.0 was used to analyze the input data into the computer. Quantitative variables that were regularly distributed were correlated using the Pearson coefficient, and regression was utilized to identify the most independent or influencing factors. NFFPS-30, SAAS, and WHO. The 5% level was used to assess the results' significance.

## Results

### Personal and academic data of nursing students (*n* = 1021)

The study had 1,021 participants, with a mean score of 19.9 years. Two-thirds (63.2%) of the nursing students were between 19 and 20 years old. Two-thirds (66.4%) were female and in their second academic year (59.8%). Regarding social relationships, (77.2%) of the participants rated their relationships as either "very good" or "excellent." Academically, 86.3% of the participants rated their performance as "very good" or "excellent," but the majority (84.2%) reported suffering from academic stress. Three-quarters (78.0%) had a family income considered "enough." Concerning health, most students (83.2%) had a normal BMI (see Table 2).

**Table 2:** Number and frequency distribution of the general characteristics of the adolescents (*n* = 1021)

Sociodemographic characteristics	n	%
**Age (Years)**		
< **19**	103	10.1
**19 – 20**	645	63.2
**21 – 22**	235	23.0
**23 – 24**	38	3.7
**Mean ± SD**	19.9 ± 1.2	
**Gender**		
**Male**	343	33.6
**Female**	678	66.4
**Academic level**		
**1st**	275	26.9
**2nd**	611	59.8
**3rd**	63	6.2
**4th**	72	7.1
**Relationship with people**		
**Weak**	13	1.3
**Acceptable**	179	17.5
**Good**	41	4.0
**Very good**	388	38.0
**Excellent**	400	39.2
**Academic performance**		
**Weak**	6	0.6
**Acceptable**	18	1.8
**Good**	116	11.4
**Very good**	458	44.9
**Excellent**	423	41.4
**Are you suffering from academic stress?**		
**No**	161	15.8
**Yes**	860	84.2
**Family income**		
**Not enough**	225	22.0
**Enough**	796	78.0
**BMI Range**		
**Normal range**	849	83.2
**Overweight**	135	13.2
**Obese**	37	3.6
**Number of times to repeat exercise per month**		
**Never**	460	45.1
**Less than 5**	367	35.9
**5 – 10**	81	7.9
**More than 10**	113	11.1

### The novo-psych five factor personality scale, the Social Anxiety About Appearance Scale (SAAS), and the WHO Quality of Life Scale-Brief (WHOQOL-Brief) score among nursing students

The trait with the highest mean score is agreeableness (21.0 ± 2.8), suggesting that respondents generally score higher on this trait with relatively low variability. Openness to experience follows closely (20.5 ± 3.6) with slightly higher variability. Conscientiousness (20.2 ± 3.1) and Extraversion (19.8 ± 3.1) have similar mean scores and moderate variability. Neuroticism has the lowest mean score (18.4 ± 4.3).

The Social Anxiety About Appearance Scale (SAAS) results show that the majority of students (79.5%) experience low levels of social anxiety, with total mean scores of 29.24 ± 14.30. Lastly, the WHO Quality of Life Scale-Brief indicates a total mean score of 244.6 with a standard deviation 59.2. The general health and social domains scored the highest in mean (58.2 and 53.2, respectively), suggesting a relatively high quality of life in social aspects compared to the physical domain, which scored the lowest at 40.6 (see Table 3).

**Table 3:** The novo-psych five factor personality scale, the Social anxiety about appearance Scale (SAAS), The WHO Quality of Life Scale-Brief (WHOQOL-Brief) total scores (*n* = 1021)

Variables	Total score Mean ± SD	
• The Novo-Psych Five Factor Personality Scale		
Openness	20.5 ± 3.6	
Conscientiousness	20.2 ± 3.1	
Extraversion	19.8 ± 3.1	
Agreeableness	21.0 ± 2.8	
Neuroticism	18.4 ± 4.3	
• The Social Anxiety About Appearance Scale (SAAS)	**29.24 ± 14.30**	
Level of SAAS	**No**	**%**
Low	802	78.6
Moderate	145	14.2
High	74	7.2
• The WHO Quality of Life Scale-Brief (WHOQOL-Brief)	244.6 ± 59.2	
General health	58.2 ± 16.3	
Physical domain	40.6 ± 11.8	
Psychological domain	48.4 ± 11.9	
Social domain	53.2 ± 18.9	
Environmental domain	44.9 ± 14.9	

### The association between the general characteristics of the study sample and novo psych five factor personality scale domains among nursing students

In Table 4**,** gender differences in personality traits are evident. Males tend to score higher on extraversion (*P* < 0.001), while females score higher on openness (*P* < 0.001), agreeableness (*P* = 0.011), and neuroticism (*P* < 0.001). Additionally, academic performance significantly affects conscientiousness (*P* < 0.001) and openness (*P* = 0.007), with higher-performing students scoring better in these areas. Poor quality of relationships correlates with lower scores in agreeableness, extraversion, and conscientiousness (all P < 0.001). Academic stress significantly influences conscientiousness, agreeableness, and neuroticism, with stressed individuals showing lower conscientiousness and higher neuroticism (*P* < 0.001). Furthermore, BMI and exercise frequency also show significant relationships with conscientiousness and neuroticism, underscoring the potential impact of physical health and lifestyle on personality traits.

**Table 4:** Association between the general characteristics of the study sample and novo psych five factor personality scale domains

General characteristics	Openness to Experience	Conscientious	Extraversion	Agreeableness	Neuroticism
	**Mean ± SD**	**Mean ± SD**	**Mean ± SD**	**Mean ± SD**	**Mean ± SD**
**Age (Years)**					
< **19**	20.3 ± 3.9	20.2 ± 3.1	19.4 ± 3.2	21.0 ± 2.8	18.5 ± 4.5
**19 – 20**	20.5 ± 3.6	20.2 ± 3.1	20.0 ± 3.1	21.1 ± 2.9	18.3 ± 4.3
**21 – 22**	20.4 ± 3.4	20.3 ± 3.3	19.5 ± 3.2	20.8 ± 2.6	18.6 ± 4.1
**23 – 24**	21.1 ± 3.1	20.3 ± 3.6	20.9 ± 2.9	21.2 ± 3.0	17.9 ± 4.3
**Oneway ANOVA**	F = 0.547, *P* = 0.650	F = 0.060, *P* = 0.981	F = 2.976, *P* = 0.031	F = 0.786, *P* = 0.502	F = 0.337, *P* = 0.798
**Gender**					
**Male**	19.5 ± 3.5	20.5 ± 3.3	20.5 ± 3.2	20.7 ± 2.7	17.2 ± 4.5
**Female**	21.0 ± 3.5	20.1 ± 3.1	19.5 ± 3.1	21.2 ± 2.9	19.0 ± 4.0
**Student’s T – Test**	T = 6.536, *P* < 0.001**	T = 1.741, *P* = 0.082	T = 4.505, *P* < 0.001**	T = 2.559, *P* = 0.011*	T = 6.714, *P* < 0.001**
**Academic level**					
**1st**	20.2 ± 3.8	20.2 ± 3.1	19.9 ± 3.1	20.9 ± 3.0	18.3 ± 4.5
**2nd**	20.6 ± 3.5	20.3 ± 3.1	19.9 ± 3.2	21.1 ± 2.8	18.3 ± 4.2
**3rd**	20.1 ± 3.5	19.8 ± 3.3	19.4 ± 3.0	20.3 ± 2.5	18.9 ± 3.7
**4th**	21.1 ± 3.6	20.0 ± 3.8	19.7 ± 3.3	21.3 ± 2.8	18.6 ± 4.0
**Oneway ANOVA**	F = 1.706, *P* = 0.164	F = 0.871, *P* = 0.456	F = 0.564, *P* = 0.639	F = 1.822, *P* = 0.141	F = 0.366, *P* = 0.778
**Relationship with people**					
**Weak**	22.4 ± 4.1	17.9 ± 4.3	17.9 ± 3.7	20.1 ± 3.3	22.2 ± 3.4
**Acceptable**	20.4 ± 3.1	19.3 ± 2.9	18.8 ± 2.8	20.7 ± 3.0	20.1 ± 4.0
**Good**	19.1 ± 4.2	18.0 ± 3.6	16.9 ± 3.9	19.5 ± 3.7	20.6 ± 4.1
**Very good**	20.6 ± 3.4	20.1 ± 2.8	19.6 ± 2.8	20.9 ± 2.7	18.8 ± 3.7
**Excellent**	20.5 ± 3.8	21.1 ± 3.2	21.0 ± 3.1	21.4 ± 2.7	16.8 ± 4.4
**Oneway ANOVA**	F = 2.532, *P* = 0.039*	F = 19.915, *P* < 0.001**	F = 31.956, *P* < 0.001**	F = 6.150, *P* < 0.001**	F = 30.329, *P* < 0.001**
**Academic performance**					
**Weak**	20.2 ± 3.7	17.3 ± 3.1	20.5 ± 2.9	20.3 ± 2.4	18.7 ± 4.2
**Acceptable**	19.6 ± 4.1	19.1 ± 3.3	19.3 ± 3.6	22.2 ± 2.8	19.6 ± 4.7
**Good**	20.0 ± 3.5	19.6 ± 3.1	19.5 ± 2.8	20.7 ± 2.9	19.0 ± 3.9
**Very good**	20.2 ± 3.6	20.0 ± 3.1	19.9 ± 3.2	21.0 ± 2.6	18.4 ± 4.1
**Excellent**	21.0 ± 3.6	20.7 ± 3.2	19.9 ± 3.2	21.1 ± 3.0	18.2 ± 4.4
**Oneway ANOVA**	F = 3.536, *P* = 0.007*	F = 6.257, *P* < 0.001**	F = 0.570, *P* = 0.685	F = 1.504, *P* = 0.199	F = 1.143, *P* = 0.335
**Are you suffering from academic stress?**					
**No**	20.3 ± 3.5	21.6 ± 3.2	20.0 ± 3.4	21.0 ± 2.7	15.5 ± 4.5
**Yes**	20.5 ± 3.6	20.0 ± 3.1	19.8 ± 3.1	21.0 ± 2.8	18.9 ± 4.0
**Student’s T – Test**	T = 0.849, *P* = 0.396	T = 5.971, *P* < 0.001**	T = 0.594, *P* = 0.553	T = 0.243, *P* = 0.808	T = 9.871, *P* < 0.001**
**Family income**					
**Not enough**	20.4 ± 3.7	19.8 ± 3.2	20.0 ± 3.4	21.0 ± 3.0	19.2 ± 4.5
**Enough**	20.5 ± 3.6	20.4 ± 3.1	19.8 ± 3.1	21.0 ± 2.8	18.2 ± 4.2
**Student’s T – Test**	T = 0.208, *P* = 0.835	T = 2.320, P = 0.021*	T = 0.711, P = 0.477	T = 0.187, P = 0.852	T = 3.330, *P* < 0.001**
**BMI Range**					
**Normal range**	20.5 ± 3.5	20.4 ± 3.1	19.9 ± 3.1	21.0 ± 2.8	18.1 ± 4.2
**Overweight**	20.1 ± 3.6	19.3 ± 2.9	19.5 ± 3.2	21.1 ± 2.9	19.3 ± 4.2
**Obese**	20.6 ± 4.7	19.1 ± 3.7	19.2 ± 3.8	21.5 ± 2.9	20.8 ± 4.9
**Oneway ANOVA**	F = 1.092, P = 0.336	F = 10.104, *P* < 0.001**	F = 1.825, P = 0.162	F = 0.637, P = 0.529	F = 11.156, *P* < 0.001**
**Number of times to repeat exercise per month**					
**Never**	20.4 ± 3.6	19.6 ± 3.1	19.3 ± 3.2	21.0 ± 2.9	19.5 ± 3.8
**Less than 5**	20.6 ± 3.5	20.8 ± 3.1	20.1 ± 2.9	21.2 ± 2.7	17.6 ± 4.1
**5 – 10**	20.2 ± 3.6	20.4 ± 3.2	20.3 ± 3.1	20.8 ± 3.0	17.8 ± 4.6
**More than 10**	20.4 ± 3.9	21.0 ± 3.1	20.9 ± 3.1	20.6 ± 2.8	16.7 ± 4.8
**Oneway ANOVA**	F = 0.451, P = 0.717	F = 13.436, *P* < 0.001**	F = 10.035, *P* < 0.001**	F = 1.287, P = 0.278	F = 23.789, *P* < 0.001**

^*^Statistically significant at *p* ≤ 0.05

** *P* ≤0.01

### Correlation between social appearance anxiety scale and who quality of life scale with novo psych five factor personality scale domains

Table 5 displays the results of the Social Appearance Anxiety Scale (SAAS). This scale shows significant negative correlations with openness (*r* = −0.114, *p* < 0.001), conscientiousness (*r* = −0.330, *p* < 0.001), and extraversion (*r* = −0.187, *p* < 0.001). There is a strong positive correlation between social anxiety and neuroticism (*r* = 0.437, *p* < 0.001). No significant correlation is found with agreeableness.

**Table 5:** Correlation between social appearance anxiety scale and who quality of life scale with novo psych five factor personality scale domains (*n* = 1021)

Variable	Openness to Experience		Conscientious		Extraversion		Agreeableness		Neuroticism	
	**r**	**P**	**r**	**p**	**r**	**p**	**r**	**p**	**r**	**P**
**Social Appearance Anxiety Scale**	−0.114	< 0.001**	−0.330	< 0.001**	−0.187	< 0.001**	−0.013	00.668	0.437	< 0.001**
**WHO Quality of Life Scale**	0.167	< 0.001**	0.401	< 0.001**	0.228	< 0.001**	0.112	< 0.001**	−0.486	< 0.001**

** *P* ≤0.01

In contrast, the WHO Quality of Life Scale demonstrates significant positive correlations with openness (*r* = 0.167, *p* < 0.001), conscientiousness (*r* = 0.401, *p* < 0.001), extraversion (*r* = 0.228, *p* < 0.001), and agreeableness (*r* = 0.112, *p* < 0.001). Conversely, there is a strong negative correlation with neuroticism (*r* = −0.486, *p* < 0.001).

### Linear regression analysis showing factors social appearance anxiety scale

The model explains 22.2% of the variance in social appearance anxiety (*R*^2^ = 0.222, Adjusted *R*^2^ = 0.218, *F* = 57.780, *p* < 0.001). Significant predictors include neuroticism, which has the strongest positive association with anxiety (B = 1.157, Beta = 0.344, *p* < 0.001), indicating that higher levels of neuroticism are linked to increased social appearance anxiety. Conversely, conscientiousness (B = −0.636, Beta = −0.139, *p* < 0.001), extraversion (B = −0.462, Beta = −0.101, *p* < 0.001), and openness (B = −0.236, Beta = −0.059, *p* = 0.038) show negative associations, suggesting that individuals with higher levels of these traits are less likely to experience social appearance anxiety (see Table 6).

**Table 6:** Linear regression analysis showing factors social appearance anxiety scale (*n* = 1021)

Variable	B	Beta	t	p	95% CI	
					**LL**	**UL**
**Openness**	−0.236	−0.059	−2.075	0.038*	−0.460	−0.013
**Conscientiousness**	−0.636	−0.139	−4.311	< 0.001**	−0.925	−0.346
**Extraversion**	−0.462	−0.101	−3.590	< 0.001**	−0.714	−0.209
**Agreeableness**	−1.193	−0.338	1.340	< 0.001**	−0.089	0.475
**Neuroticism**	1.157	0.344	10.693	< 0.001**	0.945	1.369
**R**^**2**^** = 0.222, Adjusted R**^**2**^ = 0.218,,F = 57.780^*^,*p* < 0.001^*^						

F,p: f and p values for the model

R^2^: Coefficient of determination

B: Unstandardized Coefficients

Beta: Standardized Coefficients

t: t-test of significance

LL: Lower limit UL: Upper Limit

^*^Statistically significant at *p *≤ 0.05

** *P* ≤0.01

### Linear regression analysis showing factors who quality of life scale-brief scale (*n* = 1021)

Table 7 shows that the model explains 38.7% of the variance in quality of life (*R*^2^ = 0.387, Adjusted *R*^2^ = 0.383, *F* = 106.657, *p* < 0.001). Neuroticism strongly impacts QoL (B = −3.365, Beta = −0.242, *p* < 0.001), indicating that higher neuroticism is associated with poorer quality of life. In contrast, conscientiousness (B = 2.575, Beta = 0.136, *p* < 0.001), extraversion (B = 1.820, Beta = 0.097, *p* < 0.001), openness (B = 1.076, Beta = 0.065, *p* = 0.010), and agreeableness (B = 1.268, Beta = 0.061, *p* = 0.017) all positively contribute to better quality of life. Additionally, social appearance anxiety negatively affects QoL (B = −1.403, Beta = −0.339, *p* < 0.001), showing that higher anxiety about appearance is linked to lower quality of life.

**Table 7:** Linear regression analysis showing factors WHO quality of life scale-brief scale (*n* = 1021)

Variable	B	Beta	t	p	95% CI	
					**LL**	**UL**
**Openness**	1.076	0.065	2.565	0.010*	0.253	1.900
**Conscientiousness**	2.575	0.136	4.707	< 0.001**	1.501	3.648
**Extraversion**	1.820	0.097	3.826	< 0.001**	0.887	2.753
**Agreeableness**	1.268	0.061	2.399	0.017*	0.231	2.306
**Neuroticism**	−3.365	−0.242	−8.022	< 0.001**	−4.189	−2.542
**The Social anxiety about appearance Scale (SAAS)**	−1.403	−0.339	−12.162	< 0.001**	−1.630	−1.177
**R**^**2**^** = 0.387, Adjusted R**^**2**^ = 0.383, F = 106.657^*^, *p* < 0.001^*^						

F,p: f and p values for the model

R^2^: Coefficient of determination

B: Unstandardized Coefficients

Beta: Standardized Coefficients

t: t-test of significance

LL: Lower limit UL: Upper Limit

^*^Statistically significant at *p* ≤ 0.05

** *P* ≤0.01

## Discussion

This study aimed to examine the relationships between personality traits, social appearance anxiety, and quality of life among nursing students to identify significant predictors and potential areas for intervention to enhance their overall well-being.

In line with Q1 (What is the relationship between personality traits and social appearance anxiety among nursing students?), the findings revealed that neuroticism was positively correlated with the Social Appearance Anxiety Scale (SAAS) scores, indicating that individuals with higher neurotic tendencies are more likely to experience heightened anxiety regarding their appearance (*r* = 0.437, *p* < 0.001). These results are consistent with the findings of Kong et al. [22], who also reported a positive association between neuroticism and appearance-related anxiety. Similarly, studies by Lai et al. [23] and Davis et al. [24] confirmed that neuroticism is related to appearance orientation, suggesting that individuals scoring higher in neuroticism show greater preoccupation with their appearance. Furthermore, neurotic individuals tend to exhibit emotional instability, including aggression, mood swings, and anxiety; they are prone to using maladaptive coping strategies, such as avoidance and self-blame, in high-pressure environments. These characteristics make neuroticism a strong predictor of adverse emotional outcomes. In alignment with these findings, Abbasi et al. [25] demonstrated that female students with heightened appearance anxiety reported elevated levels of neuroticism. Likewise, Contractor et al. [26] found a moderate positive correlation between neuroticism and social appearance anxiety, indicating that individuals scoring high on neuroticism are more likely to exhibit higher levels of social appearance-related anxiety. Additionally, ÇİFtÇİ et al. [12] highlight that subpersonality traits, including extraversion and neuroticism, play a significant role in shaping social appearance anxiety. However, the study also identified a negative correlation between certain personality traits, such as extraversion, emotional inconsistency, negative valence, and social anxiety levels [27].

The present study identified a statistically significant negative correlation between extraversion and openness to experience with social appearance anxiety. These findings are consistent with those reported by Abbasi et al. [25], who found that female students with elevated levels of appearance-related anxiety exhibited lower levels of extraversion and openness. Similarly, research conducted on individuals undergoing aesthetic surgery by Atasoy et al. [28] revealed moderate negative correlations between extraversion and social appearance anxiety levels.

Moreover, these findings align with those of Contractor et al.(2018) [26], who noted that individuals scoring low on the extraversion scale (i.e., introverted individuals) are more prone to experiencing social appearance anxiety. Introverted individuals feel less comfortable in social interactions and are less inclined to engage in group activities or public performances. This avoidance of social situations may further exacerbate social appearance anxiety, leading to negative correlations between extraversion and anxiety levels. Martin [29] supports this idea, suggesting that introverted individuals may avoid public settings, thus intensifying concerns about appearance in social contexts. However, inconsistent findings have been reported in some studies. For instance, Öztürk et al. [30] found no significant relationship between social appearance anxiety and the traits of openness to experience or responsibility among university students majoring in sports sciences. These discrepancies could stem from demographic differences in study populations, variations in measurement tools used to assess personality traits and anxiety levels, and the influence of cultural norms and societal expectations on personality development and appearance-related concerns.

The findings related to Q2 (How do different personality traits impact the quality of life of nursing students?) demonstrated that total quality of life (QOL) was negatively correlated with neuroticism, aligning with previous research highlighting the vulnerability of certain personality traits to stress and poorer mental health. Studies by Bunevicius et al. [31], Matsudaira et al. [32], and Steunenberg et al. [33] reported that individuals with higher levels of neuroticism tend to experience more significant stress and diminished mental well-being. More specifically, research on medical students found that pronounced levels of neuroticism increased the risk of depression and suicidal ideation [33, 34]. These findings suggest that neuroticism, as a personality trait, substantially negatively impacts QOL. In contrast, extraversion was associated with better QOL outcomes, while neuroticism was consistently linked to poorer QOL across multiple studies [35–37]. These results indicate that personality plays a crucial role in shaping life satisfaction and overall well-being, with greater extraversion contributing positively and heightened neuroticism negatively impacting QOL scores. One potential explanation for the negative association between neuroticism and QOL is related to the pessimistic and distrustful nature of individuals high in neuroticism. Such individuals may struggle to recognize the positives in life situations, leading to dissatisfaction and reduced well-being. This aligns with the findings of Abdullahi et al. [38], who noted that people with high neuroticism tend to have a negative outlook on life, contributing to poor QOL. The results of the current study indicated that extraversion is positively correlated with quality of life (QOL). This suggests that individuals high in extraversion tend to engage in activities that promote well-being and satisfaction. These findings, however, contradict previous studies such as Abdullahi et al.(2020) [38], which reported only a weak positive association between extraversion and psychological well-being. This discrepancy may be attributed to differences in the tools used in the studies to assess personality traits and QOL. Additionally, the positive association found in the current study may reflect the core characteristics of extroverted individuals—including high energy, sociability, enthusiasm, assertiveness, and emotional expressiveness. Extraverted individuals are more likely to assume leadership roles and engage actively in social environments, enhancing their satisfaction and fulfillment and contributing to better QOL [39].

On the other hand, the study also found a positive correlation between openness to experience and QOL (*r* = 0.167, *p* < 0.001). These findings contrast with Maalouf et al. [40], who reported that openness to experience negatively impacted the QOL of medical students. However, the current results align with findings by Carrillo et al. [41], who noted that openness to action is a significant predictor of psychological well-being, as it was negatively correlated with both depression and anxiety. Similarly, in Balyan et al. [42] study on the relationship between personality traits and life satisfaction, a positive relationship was observed between life satisfaction and extraversion, openness to experience, and responsibility. At the same time, emotional instability (neuroticism) was negatively associated with life satisfaction. The positive relationship between openness to experience and QOL in the present study may reflect the individual tendencies of open-minded people to pursue a wide range of life experiences, thereby fostering low anxiety levels and a greater sense of well-being [38]. Additionally, people with high openness tend to focus less on interpersonal concerns and are more inclined toward personal exploration, allowing them to experience fulfillment independently of social validation. This openness to diverse experiences could serve as a protective factor, enhancing QOL by reducing emotional distress and promoting psychological resilience.

About Q3 (Is there a significant relationship between social appearance anxiety and the quality of life among nursing students?) The present study found a negative correlation between total quality of life (QOL) and social appearance anxiety among nursing students. This result aligns with the findings of Hussain et al. [43], who reported that heightened social appearance anxiety and psychological distress contribute to a lower QOL. Individuals experiencing anxiety about their appearance often exhibit a strong desire to avoid being observed, which can intensify feelings of loneliness and exacerbate their fear of rejection due to perceived appearance flaws, leading to diminished well-being [44]. Similarly, Moneva et al. [45]found that students are more likely to encounter appearance-related anxiety in social contexts, reinforcing the negative impact of such anxieties on their interpersonal relations and well-being. However, some research presents conflicting findings. For example, ÇİFtÇİ et al. [12]reported no significant relationship between social appearance anxiety and life satisfaction, highlighting the complexity of this relationship across different populations and contexts.

The current study further revealed that all subdomains of QOL—physical, psychological, social, and environmental—were significantly negatively correlated with social appearance anxiety (*p* < 0.001). This suggests that social appearance anxiety affects multiple dimensions of well-being. These findings are consistent with Atasoy et al. [46], who noted that social appearance concerns reflect how individuals perceive others, evaluate their physical appearance, and influence self-esteem and QOL. Harter [47] emphasized that physical respect is a key factor shaping self-esteem, which, in turn, profoundly impacts overall QOL.

Additionally, Bektaş et al. [48] highlighted that QOL encompasses the totality of an individual's emotions, thoughts, and levels of consciousness based on their subjective assessment of life experiences. Consequently, anxiety in social situations—particularly related to physical appearance can directly influence QOL by diminishing emotional stability and satisfaction [46].

## Conclusion

All personality traits (extraversion, openness to experience, and consciousness) are significantly negatively correlated with SAAS except neuroticism, which was positively correlated. However, agreeableness is not significantly correlated with SASS. Quality of life was positively correlated with all subtypes of personality except neuroticism. SAAS and neuroticism personality traits are strong negative predictors of quality of life.

### Implications

Addressing the association between the studied variables in nursing students will give new perspectives on managing many psychosocial and educational problems that nursing students could face, negatively affecting their academic performance and clinical competence. Educational programs for nursing students on evidence-based studies will help manage their academic difficulties and enable them to engage effectively with patients during clinical practice. Investigate the long-term effects of SAAS and personality traits on the transition from nursing education to professional practice, focusing on career satisfaction, patient care quality, and mental health outcomes. Promoting self-awareness through workshops can help students manage neuroticism and enhance traits like extraversion and openness to improve quality of life (QOL). Counseling and support groups can enhance confidence and clinical skills while integrating personality-focused and mental health programs into the nursing curriculum, which can reduce stress and social appearance anxiety, foster resilience, and support long-term professional success. These strategies will enhance academic achievement and competent healthcare professionals' development. Future studies could adopt a longitudinal approach to explore how personality traits and social appearance anxiety (SAAS) evolve during nursing education, particularly students’ progress through different academic years and clinical training stages. Another study needs to be made on the effect of personality on academic stress and its relation to quality of life among nursing students.

### Limitations

Our research presents several notable limitations that must be acknowledged. Firstly, the questionnaire was considerably lengthy. As a result, some students needed help completing their questionnaires fully, which led to excluding those instances from our analysis. This exclusion hindered our ability to obtain a uniform and representative sample from each academic year, making it challenging to analyze variations in critical variables such as students' personality characteristics, levels of social appearance anxiety, and overall quality of life.

Furthermore, the intricate and multifaceted relationships among personality traits, social appearance anxiety, and quality of life present additional complexities. This study's cross-sectional design limits our understanding of these relationships. Conducting longitudinal studies to gain a more precise and comprehensive understanding of how these factors interact is crucial.

## Data Availability

The datasets used during the study are available from the corresponding author upon request.

## Abbreviations

NFFPS: Novo Psych Five Factor Personality Scale
SAAS: Social Anxiety about Appearance Scale
WHOQOL-Brief: WHO Quality of Life Scale-Brief

## References

[1] Shahzadi K. Personality Traits, Social Anxiety and Social Support among Adults. World Journal of Pharmaceutical and Medical Research. 2024;10(5):23–41.

[2] Holzman PS, Proctor LR, Levy DL, Yasillo NJ, Meltzer HY, Hurt SW. Eye-tracking dysfunctions in schizophrenic patients and their relatives. Arch Gen Psychiatry. 2019;31(2):143–51. 10.1001/archpsyc.1974.01760140005001 4851993

[3] Khorsandi F, Kamkar M, Malakpour M. The relationship between the five-factor personality traits and self-regulated learning strategies in male and female high school students from 2007–2008]. New educational approaches. 2010;5(2):41–64.

[4] Vala R, Jamaliarani M. The relationship between personality traits and religious beliefs. Insight and Islamic training. 2014;11(29):83–111.

[5] Latifi S, Allah Karami A, Baba Moradi A. Predicting teachers' computer anxiety based on their personality traits and emotional intelligence components. Infirm Commun Tech Educ Sci. 2014;4(2 (14)):131–48.

[6] Majeed S, Munir M, Malik K. Academic self-efficacy, social anxiety and academic success in university students. Pakistan Languages and Humanities Review. 2022;6(3):69–81.

[7] Yuceant M, Unlu H. The analysis of social appearance anxiety levels of physical education teacher candidates in terms of different variables. Turkish Journal of Sport and Exercise. 2017;19(1):102–8.

[8] Claes L, Hart TA, Smits D, Van den Eynde F, Mueller A, Mitchell JE, et al. Validation of the Social Appearance Anxiety Scale in female eating disorder patients. Eur Eat Disord Rev. 2012;20(5):406–9. 21805536 10.1002/erv.1147

[9] Antonietti C, Camerini A-L, Marciano L. The impact of self-esteem, family and peer cohesion on social appearance anxiety in adolescence: an examination of the mediating role of coping. Int J Adolesc Youth. 2020;25(1):1089–102.

[10] Cetin S, Ece C. Investigation of Social Appearance Anxiety in University Students. Pakistan Journal of Medical and Health Sciences. 2021;15(5):1694–8.

[11] WHO. WHOQOL - Measuring Quality of Life| The World Health Organization. [cited 2025 Jan 7]. Available from: https://www.who.int/tools/whoqol.

[12] ÇİFtÇİ İ, ÇAkir VO. The Effect of Personality Factors on Social Appearance Anxiety and Life Satisfaction. In J Disabil Sports Health Sci. 2023:169–77.

[13] Nayir T, Uskun E, Yürekli MV, Devran H, Çelik A, Okyay RA. Does Body Image Affect Quality of Life?: A Population-Based Study. PLoS One. 2016;11(9):e0163290-e. 10.1371/journal.pone.0163290 PMC5029906 27649389

[14] Grogan S. Body Image: Routledge; 2021 2021/10/14.

[15] Swami V, Horne G, Furnham A. COVID-19-related stress and anxiety are associated with negative body image in adults from the United Kingdom. Pers Individ Differences. 2021;170:110426-. 10.1016/j.paid.2020.110426 PMC7539826 33046945

[16] Liao J, Xia T, Xu X, Pan L. The Effect of Appearance Anxiety on Social Anxiety among College Students: Sequential Mediating Effects of Self-Efficacy and Self-Esteem. Behav Sci (Basel). 2023;13(8):692. 37622832 10.3390/bs13080692 PMC10451712

[17] Shakir W, Tariq M, Khalid F, Sahar. Self-esteem, Social Appearance Anxiety and Quality of Life among Adolescents with Acne. J Health Rehabil Res. 2024;4(2):577–84.

[18] Buchanan B, Hegarty D. Development of a short personality assessment: The NovoPsych Five Factor Personality Scale – 30-item version. NovoPsych. 2023. https://novopsych.com.au/wp-content/uploads/2023/09/NFFPS-30-article-NovoPsych-website-version.pdf.

[19] Hart TA, Flora DB, Palyo SA, Fresco DM, Holle C, Heimberg RG. Development and Examination of the Social Appearance Anxiety Scale. Assessment. 2008;15(1):48–59. 18258731 10.1177/1073191107306673

[20] Mills SD, Kwakkenbos L, Carrier ME, Gholizadeh S, Fox RS, Jewett LR, et al. Validation of the Social Appearance Anxiety Scale in patients with systemic sclerosis: a scleroderma patient-centered intervention network cohort study. Arthritis Care Res. 2018;70(10):1557–62. 10.1002/acr.23514 29342510

[21] WHO. WHOQOL: Measuring Quality of Life. World Health Organization. 1997.

[22] Kong X, Cao Y, Luo X, He L. The correlation analysis between the appearance anxiety and personality traits of the medical staff on nasal and facial pressure ulcers during the novel coronavirus disease 2019 outbreak. Nurs Open. 2020;8(1):147–55. 33318822 10.1002/nop2.613 PMC7729537

[23] Lai F, Wang L, Zhang J, Shan S, Chen J, Tian L. Relationship between Social Media Use and Social Anxiety in College Students: Mediation Effect of Communication Capacity. Int J Environ Res Public Health. 2023;20(4):3657. 36834357 10.3390/ijerph20043657 PMC9966679

[24] Davis C, Karvinen K, McCreary DR. Personality correlates of a drive for muscularity in young men. Personality Individ Differ. 2005;39(2):349–59.

[25] Abbasi F, Tabatabaee-Yazdi M. EFL teachers’ personality traits and sense of technophobia and technophilia. Journal of Research in Techno-based Language Education. 2021;1(2):1–14.

[26] Contractor R, Sarkar S. Understanding the Relationship between Social Anxiety and Personality. Indian J Mental Health(IJMH). 2018;5(1):107.

[27] Uçar MZ. The Relationship Between Personality Traits and Psychological Resilience Levels of Adolescent Individuals. Konya in Turkish: Selçuk University; 2020.

[28] Atasoy T, Pekel A. The relationship between quality of life level and social appearance anxiety level of physically handicapped boccia athletes. Phys Ed. 2021;78(1):1–0.

[29] Martin CR. Looking at type: The fundamentals: Center for Applications of Psychological Type; 2010.

[30] Öztürk A, Kara A, Körük S. The Predictive Power of University Students’ Personality Characteristics, Gender Roles, and Blushing Tendency on Social Appearance Anxiety, and the Relationship between Them. Electronic Turkish Studies. 2015;10(10):1–12.

[31] Bunevicius A, Katkute A, Bunevicius R. Symptoms of Anxiety and Depression in Medical Students and Humanities Students: Relationship With Big-Five Personality Dimensions and Vulnerability To Stress. Int J Soc Psychiatry. 2008;54(6):494–501. 18974188 10.1177/0020764008090843

[32] Matsudaira T, Kitamura T. Personality traits as risk factors of depression and anxiety among Japanese students. J Clin Psychol. 2005;62(1):97–109. 10.1002/jclp.20215 16287151

[33] Steunenberg B, Beekman ATF, Deeg DJH, Kerkhof AJFM. Personality and the onset of depression in late life. J Affect Disord. 2006;92(2–3):243–51. 16545466 10.1016/j.jad.2006.02.003

[34] Tyssen R, Hem E, Vaglum P, Grønvold NT, Ekeberg Ø. The process of suicidal planning among medical doctors: predictors in a longitudinal Norwegian sample. J Affect Disord. 2004;80(2–3):191–8. 15207932 10.1016/S0165-0327(03)00091-0

[35] Steel P, Schmidt J, Shultz J. Refining the relationship between personality and subjective well-being. Psychol Bull. 2008;134(1):138–61. 18193998 10.1037/0033-2909.134.1.138

[36] DeNeve KM, Cooper H. The happy personality: A meta-analysis of 137 personality traits and subjective well-being. Psychol Bull. 1998;124(2):197–229. 9747186 10.1037/0033-2909.124.2.197

[37] Lucas RE, Fujita F. Factors influencing the relation between extraversion and pleasant effect. J Pers Soc Psychol. 2000;79(6):1039–56. 11138753 10.1037//0022-3514.79.6.1039

[38] Abdullahi AM, Orji R, Rabiu AM, Kawu AA. Personality and Subjective Well-Being: Towards Personalized Persuasive Interventions for Health and Well-Being. Online J Public Health Inform. 2020;12(1):e1-e. 10.5210/ojphi.v12i1.10335 PMC7289121 32547678

[39] Călin MF, Șerban LE. Effects of personality traits on the quality of life in adults. Int J Legal Soc Order. 2021;1(1).

[40] Maalouf E, Hallit S, Obeid S. Personality traits and quality of life among Lebanese medical students: any mediating effect of emotional intelligence? A path analysis approach. BMC Psychol. 2022;10(1):28-. 10.1186/s40359-022-00739-2 PMC8840643 35148803

[41] Carrillo JM, Rojo N, Sánchez-Bernardos ML, Avia MD. Openness to Experience and Depression* * The original data upon which this paper is based are available atwww.hhpub.com/journals/ejpa*.* Eur J Psychol Assess. 2001;17(2):130–6.

[42] Balyan M, Köksal A, Gönkek P, Zekioğlu A, Başoğlu UD. Analysıs of Emotıonal Intellıgence and Personalıty Traıts of Students In Faculty of Sports Scıences. Propósitos y Representaciones. 2021;9(SPE3).

[43] Hussain M, Tariq M, Hussain M. Social Appearance Anxiety, Psychological Distress and Quality of Life among Patients with Burn Injuries. Journal of Professional & Applied Psychology. 2023;4(3):418–28.

[44] Papapanou TK, Darviri C, Kanaka-Gantenbein C, Tigani X, Michou M, Vlachakis D, et al. Strong Correlations Between Social Appearance Anxiety, Use of Social Media, and Feelings of Loneliness in Adolescents and Young Adults. Int J Environ Res Public Health. 2023;20(5):4296. 36901307 10.3390/ijerph20054296 PMC10001671

[45] Moneva JC, Bantasan M, Vertulfo RM. Performance Tasks and Socialization of Students with Broken Family. International Journal of Social Science Research. 2020;8(2):88.

[46] Atasoy T, Pekel A. The relationship between quality of life level and social appearance anxiety level of physically disabled boccia athletes. Physical Educator. 2021;78(1):1–10.

[47] Harter S. The development of the self-esteem. In: Hetherington, editor. Handbook of child psychology: Social and personality development. USA: Wiley; 1983.

[48] Bektaş FM, Tekeli O. Association of Quality of Life and Visual Function in Glaucoma with Tests of Structure and Function. J Glaucoma. 2024:10–97. 10.1097/IJG.0000000000002495 39259003

